# Matefin/SUN-1 Phosphorylation on Serine 43 Is Mediated by CDK-1 and Required for Its Localization to Centrosomes and Normal Mitosis in *C. elegans* Embryos

**DOI:** 10.3390/cells5010008

**Published:** 2016-02-24

**Authors:** Noam Zuela, Yosef Gruenbaum

**Affiliations:** Department of Genetics, Institute of Life Science, Hebrew University of Jerusalem, Jerusalem 91904, Israel

**Keywords:** *C. elegans*, centrosome, LINC complex, mitosis, meiosis, SUN-domain

## Abstract

Matefin/SUN-1 is an evolutionary conserved *C. elegans* inner nuclear membrane SUN-domain protein. By creating a bridge with the KASH-domain protein ZYG-12, it connects the nucleus to cytoplasmic filaments and organelles. Matefin/SUN-1 is expressed in the germline where it undergoes specific phosphorylation at its N-terminal domain, which is required for germline development and homologous chromosome pairing. The maternally deposited matefin/SUN-1 is then essential for embryonic development. Here, we show that in embryos, serine 43 of matefin/SUN-1 (S43) is phosphorylated in a CDK-1 dependent manner and is localized throughout the cell cycle mostly to centrosomes. By generating animals expressing phosphodead S43A and phosphomimetic S43E mutations, we show that phosphorylation of S43 is required to maintain centrosome integrity and function, as well as for the localization of ZYG-12 and lamin. Expression of S43E in early embryos also leads to an increase in chromatin structural changes, decreased progeny and to almost complete embryonic lethality. Down regulation of emerin further increases the occurrence of chromatin organization abnormalities, indicating possible collaborative roles for these proteins that is regulated by S43 phosphorylation. Taken together, these results support a role for phosphorylation of serine 43 in matefin/SUN-1 in mitosis.

## 1. Introduction

The nuclear envelope is composed of outer and inner nuclear membranes which fuse at nuclear pore complexes and the nuclear lamina, which is a meshwork of proteins positioned underneath the inner nuclear membrane. The nuclear lamina is composed of lamin filaments and numerous lamin-associated proteins [1]. Many of the lamin-associated proteins are integral proteins of the inner nuclear membrane [2]. Some of these integral proteins are expressed in most cells, similar to lamins, while others have tissue-specific expression [3].

Lamins are nuclear intermediate filaments and the major building blocks of the nuclear lamina. They are required to maintain nuclear structure and, together with their associated proteins, are involved in most nuclear activities including regulating the mechano response, organizing chromosomes and regulating the cell cycle, as well as regulating DNA replication and cell signaling [1]. A small fraction of lamins is also present in the nucleoplasm. This fraction is more soluble and dynamic compared to the peripheral lamins [4]. It interacts with unique sets of proteins and is involved in cell cycle progression [5]. Mammals have 3 lamin genes and four major protein products (lamins A, B1, B2 and C) that are divided into A-type and B-type lamins. Mutations in lamins cause numerous diseases termed laminopathies [6]. The *C. elegans* genome has one lamin gene (*lmn-1*) which encodes a single lamin protein that has characteristics of both A- and B-type lamins [7]. Like vertebrate B-type lamins, the *C. elegans* lamin is expressed in all cells throughout development. It remains permanently farnesylated and interacts with proteins that in vertebrates bind B-type lamins. Similar to the vertebrate A-type lamins, a small fraction of the *C. elegans* lamin is present in the nucleoplasm. It is essential for maintaining nuclear shape, required for the spatial distribution of nuclear pore complexes (NPCs), and also interacts with proteins that in vertebrates bind A-type lamins, including emerin, LEM-2 and BAF-1 [8].

The LEM-domain proteins are a family of proteins that are characterized by a ~40 residue motif [9]. Most LEM-domain proteins are integral proteins of the inner nuclear membrane that interact directly with lamins [5]. The *C. elegans* genome encodes three LEM-domain proteins, two of which are embedded in the inner nuclear membrane, termed emerin and LEM2/MAN1. These integral proteins have overlapping functions in cell cycle regulation, chromatin organization and centrosome positioning [10,11].

Most SUN-domain proteins are integral proteins of the inner nuclear membrane, where they interact with lamins. The ~120 residues SUN-domain motif is positioned in the perinuclear space between the inner and outer membrane, where it also interacts as a trimeric form with the KASH domain motif [12,13,14]. Most KASH-domain proteins are localized to the outer nuclear membrane. The interaction between SUN- and KASH-domain proteins, which forms the LINC complex (Linker of Nucleoskeleton and Cytoskeleton), is required for the cellular functions of both types of proteins [15].

The *C. elegans* genome encodes two SUN-domain proteins: UNC-84 and matefin/SUN-1 [16]. The nuclear envelope localization of UNC-84 depends on its binding to lamin [17,18]. In early embryonic stages, it is required to anchor the centrosome to the nuclear periphery. UNC-84 is also required to anchor nuclei within the hypodermal syncytium, for nuclear migration in P cells during early embryogenesis and for the migration of the two distal gonadal tip cells [17,19,20].

Matefin/SUN-1 does not depends on lamin for its nuclear envelope localization, but it can directly bind to lamin [21]. It is expressed in the germline where it is essential for germline development [21]. It is maternally deposited to the egg and this maternal fraction is essential for embryonic development, since lack of matefin/SUN-1 leads to embryonic lethality at the ~300-cell stage with defects in nuclear structure, DNA content, and chromatin morphology [21]. During germline development and differentiation, matefin/SUN-1 forms the LINC complex with the KASH-domain protein ZYG-12, and this complex transmits cytoplasmic microtubule motor forces that allow chromosomal movement and homologous pairing, while preventing nonhomologous synapsis [22]. In the meiotic transition zone, matefin/SUN-1 aggregates at sites of chromosome attachment to the nuclear envelope. In the first embryonic divisions ZYG-12 is required for centrosome attachment [23].

The formation of matefin/SUN-1 aggregates and the dynamic chromosome movements to find the homologs require phosphorylation of specific residues on matefin/SUN-1 amino terminal domain [22,24]. Specific antibodies raised against the different phospho sites were used to show that the CHK-2- and PLK-2-dependent phosphorylation occurs in leptotene/zygotene, diminish during pachytene and is involved in pairing. [22,24,25]. The phosphorylation of matefin/SUN-1 is part of a checkpoint system that is critical to delaying meiosis in response to perturbed synapsis [25]. Here, we set out to study whether these amino terminal phosphorylation events of matefin/SUN-1 are also involved in embryonic development. Our results show phosphorylation of serine 43 in the amino part of matefin/SUN-1 which is mediated by CDK-1 and required for localization and organization of centrosomes, embryonic survival and localization of lamin and ZYG-12.

## 2. Experimental Section

### 2.1. Strains

*C. elegans* strains were maintained and manipulated under standard conditions as previously described [26] *C. elegans* strains N2 and RB1276 *mtf-1/sun-1(ok1282)* were provided by the *Caenorhabditis* Genetics Center, which is funded by the NIH National Center for Research Resources. FKR-3 *mtf-1/sun-1:GFP , ∆mtf (ok1282) nT1*, 929 (matefin/SUN-1 ^−/S43E^) *(∆mtf (ok1282) nT1) - UV73 JfSi13[Psun-1::GFP(S43E) cb-unc-119(+)] II; sun-1(ok1282) V/nT1 [qIs51]* was kindly provided by Verena Jantsch. COP89 (matefin/SUN-1 ^+/S43A^) *Psun-1::GFP(S43A) cb-unc-119(+)* was created by Knudra Transgenics (Salt Lake City, UT, 84115, USA), matefin/SUN-1^−/S43A^: COP89 X RB1276, matefin/SUN-1 ^+/S43E^: 929 X N2. Mutant strains were out-crossed three times to ensure a clean background.

### 2.2. RNAi Experiments

Experiments were performed by the feeding technique, as described previously [27]. The clones were obtained from Ahringer’s RNAi library [28], and their efficiency was determined by a phenotypic analysis of embryos. The empty vector L4440 was used as a control, and the DY3.2, M01D7.6, T05G5.3, C14B9.4, F09E5.1, Y18D10A.5, T27E9.3, F55H2.6, H39E23.1, K03E5.3, B0545.1, F57F5.5 and Bm16893 vectors were used to down-regulate *lmn-1*, *emr-1*, *cdk-1*, *plk-1*, *pkc-3*, *gsk-3*, *cdk-5*, *clu-1*, *par-1*, *cdk-2*, *tpa-1*, *pkc-1* and *alt-1*, respectively. For feeding experiments, we placed bleached embryos on feeding plates at 20 °C, and early staged embryos were collected and immunostained for analysis.

### 2.3. Fecundity Experiments

Thirty gravid adult worms from each strain were bleached and the isolated embryos were grown on NGM/OP50 plates until they reached the late L4 stage. Single L4 animals were then transferred, each to a separate NGM/OP50 plate. During the first three days, each worm was transferred daily to a fresh plate, while keeping the older plates. On the third day, the worms were given 24 h to lay eggs and then discarded. The progeny was counted 48 h after the worm was removed from the plate. The progeny were scored as the mean number of laid eggs per worm. Progeny survival rate was calculated as the mean of the percentage of eggs that developed into larvae from the total eggs lay. Fecundity experiments were performed at 20 °C.

### 2.4. Indirect Immunofluorescence

#### 2.4.1. Embryo Staining

Ten *C. elegans* worms at the second day of egg laying were placed in a 9 µL drop of M9 buffer on the 0.1% poly-lysine (Sigma 0.1% w/v Cat#P8920) coated area of a slide (Super Frost/Plus, Menzelglass Cat#041300). A 40 mm long cover-slip was placed on top of the drop and gently taped with forceps in order to spread the water and to allow eggs to spill out of the worm. Worms were freeze-cracked using liquid nitrogen, than fixated in cold methanol (−20 °C) for 10 min followed by 4% paraformaldehyde (Electron Microscope Sciences, Hatfield PA, USA, Cat#15710) diluted in 1X phosphate buffered saline (PBS) containing 0.1% Tween 20 (PBST) for 10 min at room temperature. Worm containing slides were blocked in PBST solution containing 0.1% low fat milk for 15 min at room temperature. The primary antibodies were applied overnight at 4 °C. Antibodies were diluted in PBST solution containing 0.1% low fat milk as follows: anti-α-tubulin (clone DM1A, Sigma, Cat#T6199) 1:300, anti- matefin/SUN-1 pS43 1:1000, anti- matefin /SUN-1 pS8 1:700, anti- matefin /SUN-1 pS24 1:50, anti-ZYG-12 1:200, anti -ce-lamin (3932 bleed 6 or 3933 ex) 1:100, anti - matefin /SUN-1 (#2824 bleed 4, antibody against an N-terminal peptide) 1:100, anti-GFP (Roach Cat#11814460001)1:400 and anti-SPD-5 1:1000. After two 1 h washes in PBST, secondary antibodies, diluted 1:200, were applied (anti-rabbit-Cy2, anti-mouse-Cy3, anti-rat-Cy2, anti-guinea pig-Cy3 and anti-rat-Cy3) for 3 h at room temperature. After two washes in PBST, DNA was stained for 10 min at room temperature using 4′, 6-Diamidino-2-phenylindole dihydrochloride (DAPI dihydrochloride, Sigma, Cat# 28718903) diluted 1:1000 in PBST, followed by a 10 min wash in PBST. Samples were mounted in a drop of 2% N-propyl gallate in glycerol and sealed with nail polish. Stained embryos were visualized using a Hamamatsu charge-coupled device (CCD) camera mounted on a Zeiss Axioplan II microscope equipped with epifluorescence.

#### 2.4.2. Gonad Staining 

Six *C. elegans* worms a day after reaching the L4 stage were placed in a 6 µL drop of PBS containing 1mM levamisol on a 0.1% poly-lysine (Sigma 0.1% w/v Cat#P8920) coated slide (Super Frost/Plus, Menzelglass Cat#041300). Gonads were extracted from worms by cutting the head and tail regions with needles. An additional drop of 6 µL 2% PFA (Electron Microscope Sciences, Hatfield PA, USA, Cat#15710) was added. A 40 mm long cover-slip was placed on top of the drop and slides were incubated at room temperature for 10 min. Worms were freeze-cracked using liquid nitrogen, than fixated in cold methanol (−20 °C) for 2 min followed by three 5 min washes in PBST. Chromatin was stained for 10 min at room temperature using 4′, 6-Diamidino-2-phenylindole dihydrochloride (DAPI dihydrochloride, Sigma USA, Cat# 28718903) diluted 1:1000 in PBS, followed by a 10 min wash in PBST. Samples were mounted in a drop of 2% N-propyl gallate in glycerol and sealed with nail polish. Gonads were visualized using a Hamamatsu CCD camera mounted on a Zeiss Axioplan II microscope equipped with epifluorescence.

### 2.5. Molecular Cloning and Strain Creation

Matefin/SUN-1 carrying the S43A substitution fused to GFP and expressed under BAF-1 promoter was inserted to the MCS of the pCFJ151 (Addgene) between restriction enzymes XhoI and AflII. This plasmid was injected into the *C. elegans* strain EG4322 (ttTi5605 II; unc-119(ed3) III) along with the co-expression plasmid pCFJ90 and the transposase expressing pJL43.1 plasmid (Pglh-2::Transposase). A single copy insertion of the pBAF-1::matefin/SUN-1 S43A::GFP transgene was generated (Knudra Transgenics, Salt Lake City, UT 84115, USA) essentially as described in [21].

### 2.6. Analysis of Relative Nucleoplasmic Peripheral Staining of Lamin

Mutant embryos were stained for ce-lamin and DNA and subsequently imaged using a Hamamatsu CCD camera mounted on a Zeiss Axioplan II microscope equipped with epifluorescence. Using the ImageJ program, the relative nucleoplasm peripheral lamin antibody staining (indicated as grey value by ImageJ) was measured as follows: mean nucleoplasmic grey value divided by mean peripheral (nuclear envelope) grey value and normalized to the mean nucleoplasmic/peripheral average grey value of wild type matefin::GFP. Mean nuclear grey values were measured as the average nuclear grey value of the nucleus minus mean background signal. The mean nucleoplasmic grey value was measured as the average nucleoplasmic grey value minus the mean background signal. The mean peripheral grey value was measured as total nuclear grey value minus total nucleoplasmic grey value divided by nuclear area minus the nucleoplasmic area.

## 3. Results and Discussion

### 3.1. Results

#### 3.1.1. The Phosphorylated Form of Serine 43 in Matefin/SUN-1 Co-Localizes with Centrosomes

**Figure 1:**
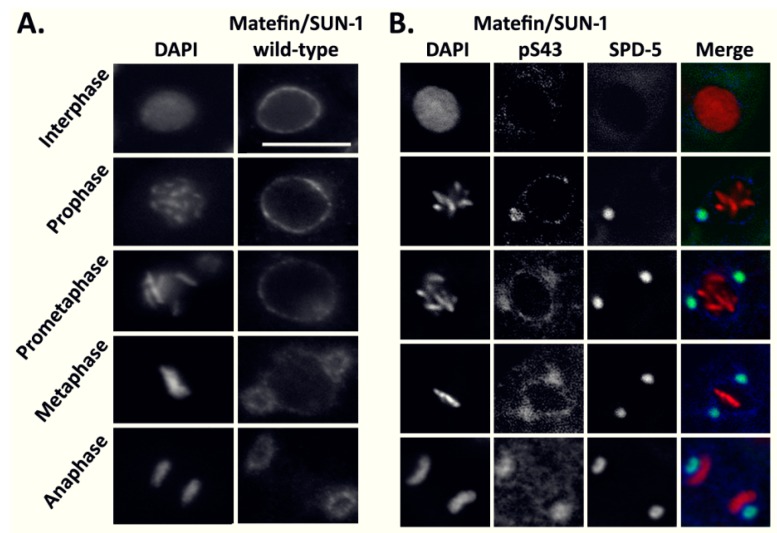
Matefin/SUN-1 pS43 localizes to centrosomes. (**A**) wild-type matefin/SUN-1 and (**B**) matefin/SUN-1 pS43 (**blue**) co-localize with the centrosome marker SPD-5 (**green**) during the different stages of cell cycle (DNA indicated in **red**). Scale bar 10 µm.

Seven phosphorylation sites of matefin/SUN-1 were previously mapped and specific antibodies were raised against these phosphorylation sites [22,25]. Since the maternally deposited matefin/SUN-1 is essential for embryonic development [21], we analyzed the phosphorylation state of this protein during consecutive cell cycle phases in early embryos. Staining *C. elegans* embryos with general anti-matefin/SUN-1 antibodies showed nuclear peripheral staining in interphase and prometaphase, and from metaphase to anaphase matefin/SUN-1 was enriched around the centrosomes, as previously described [21] (Figure 1A). Staining early *C. elegans* embryos with antibodies against phospho S8 or phospho S24 also showed nuclear peripheral staining that co-localized with lamin during interphase but persisted to the nuclear periphery until metaphase (Figure S1). In contrast, the phospho serine 43 (pS43) antibody showed specific staining only during mitosis where it was enriched at the centrosome as evident by co-localization with the centrosome marker SPD-5 (Figure 1B).

This analysis shows that phosphorylation of matefin/SUN-1 at S43 occurs in mitosis during embryogenesis, and is mostly limited to the centrosomes.

#### 3.1.2. Phosphorylation of Serine 43 in Matefin/SUN-1 Requires CDK-1 Activity

**Figure 2:**
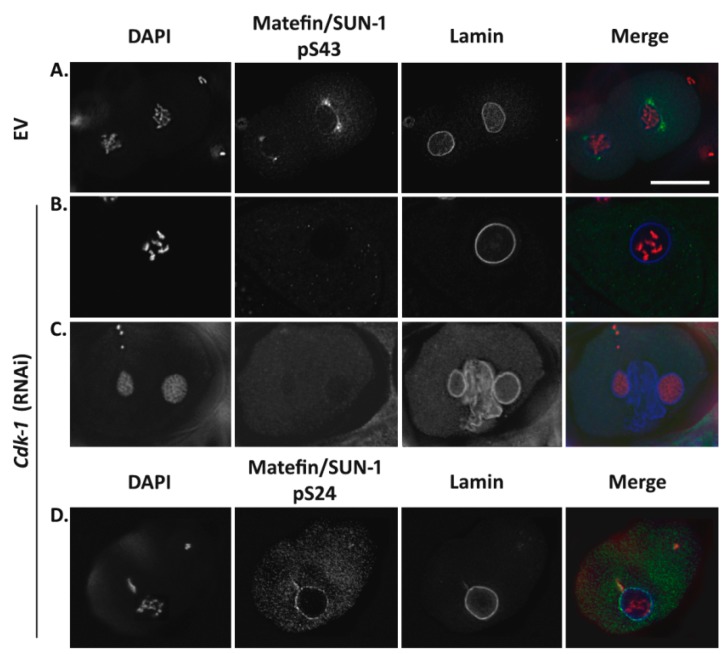
Matefin/SUN-1 pS43 is mediated by CDK-1. *cdk-1* (RNAi) specifically eliminates matefin/SUN-1 pS43 staining in embryos. (**A**) embryos treated with an empty vector (EV); (**B**–**D**) embryos treated with *cdk-1* (RNAi). Embryos stained with anti ce-lamin (blue), DAPI (**red**) and anti matefin/SUN-1 pS43 (**A**–**C**) and pS24 (**D**) (**green**) antibodies. Scale bar 10 µm.

In order to identify kinases involved in the phosphorylation of S43 in matefin/SUN-1, we analyzed embryonic development and the presence of S43 matefin/SUN-1 phosphorylation in embryos downregulated for protein kinases predicted to interact with the N-terminal tail of matefin/SUN-1, as well as several other kinases known to be involved in mitosis (Figure S2). CDK-1 is one of the kinases predicted to interact with matefin/SUN-1 N-terminal domain. It is predominantly expressed in the embryo, is involved in cell-cycle progression in both mitosis and meiosis and appears on mammalian centrosomes during prophase [29,30]. Embryos downregulated for CDK-1 displayed many DNA abnormalities as a result of the disruption in mitosis, including multiple nuclei, larger nuclei and nuclear division defects. Lamin staining revealed additional phenotypes including nuclear fusions and membrane-like structures between nuclei. These phenotypes probably resulted from improper assembly/disassembly of nuclei during the cell cycle. pS43 staining was absent in *cdk-1* (RNAi) embryos (Figure 2B,C). In contrast, other phosphorylation sites on matefin/SUN-1 were not affected (an example is shown for S24 in Figure 2D). Furthermore, downregulation of other tested kinases (e.g., *plk-1*, *pkc-3*, *gsk-3*, *cdk-5 clu-1*, *par-1*, *cdk-2*, *tpa-1*, *pkc-1* and *alt-1*) did not affect S43 phosphorylation (Figure S2). These results suggest that CDK-1 is specifically involved in matefin/SUN-1 phosphorylation of S43.

#### 3.1.3. Phosphorylation of Serine 43 in Matefin/SUN-1 Is Required to Maintain Centrosome Shape and Function

We previously showed that wild-type matefin/SUN-1 fused to GFP is fully active and can replace the endogenous matefin/SUN-1 [22]. To analyze the roles of pS43, we used the MosSCI single copy gene insertion technique [31] to generate *C. elegans* strains expressing matefin/SUN-1::GFP with the missense S43E mutation that mimics constitutive phosphorylation as well as S43A mutation, which keeps matefin/SUN-1 in a constitutive non-phosphorylated state. Each strain was also crossed with a strain null for matefin/SUN-1 *(ok1282)* [32]. In these strains, we found dramatic cellular phenotypes (see below).

**Figure 3:**
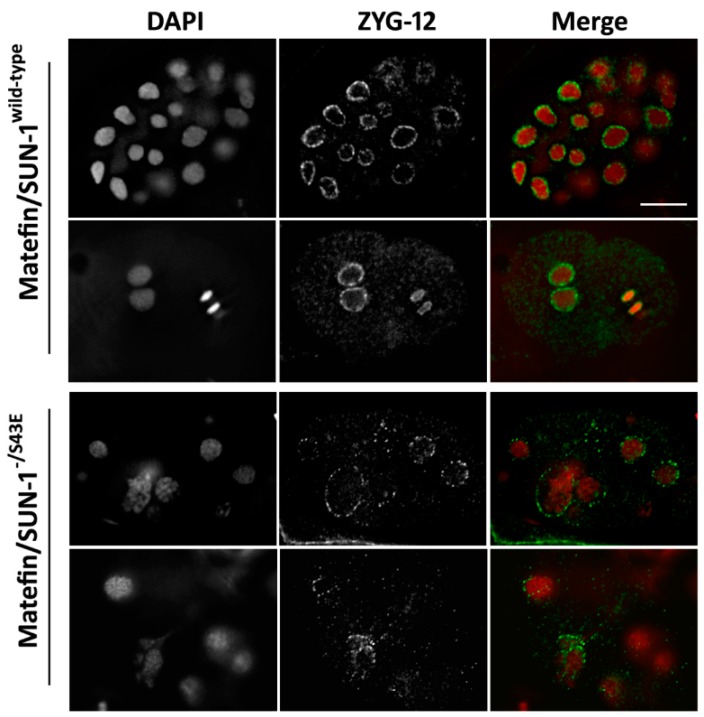
Matefin/SUN-1 pS43 mediates proper NE localization of ZYG- 12. ZYG-12 loses its NE localization in early *C. elegans* embryos expressing matefin/SUN-1 S43E. Top panels—wild-type matefin/SUN-1 embryos stained with DAPI (**red**) and anti ZYG-12 antibodies (**green**); Bottom panels—matefin/SUN-1 S43E embryos stained with DAPI (**red**) and anti ZYG-12 antibodies (**green**). Scale bar 10 µm.

**Figure 4:**
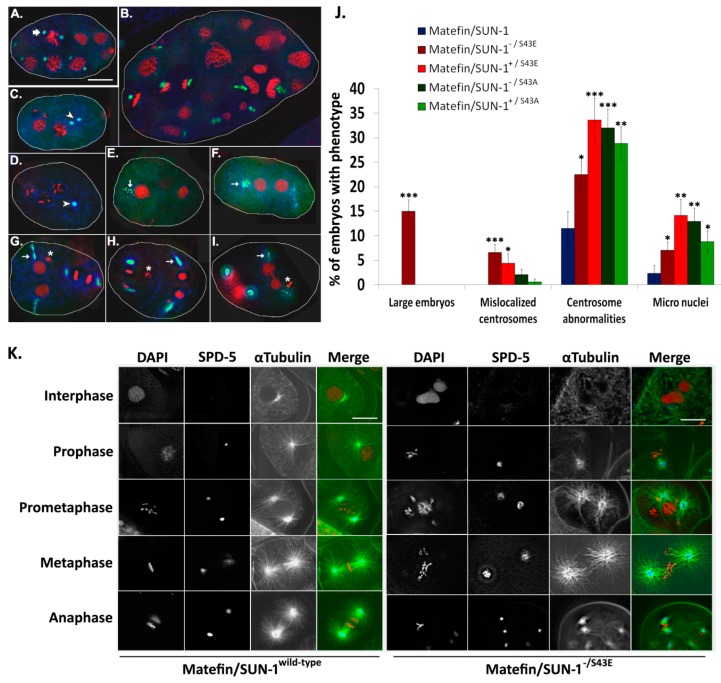
Matefin/SUN-1 S43 is involved in maintaining centrosome shape and function. DNA and centrosome phenotypes were analyzed in early *C. elegans* embryos expressing matefin/SUN-1 S43 phosphorylation mutations. (**A**–**I**) embryos stained with DAPI (**red**), anti-GFP (**blue**) and anti SPD-5 (**green**) antibodies; Phenotypes observed: Large embryos with large nuclei (**B**); mislocalized centrosomes (arrowheads **C**–**D**) centrosome abnormalities (centrosome break-down (arrows **E**,**F**) and centrosome stretch (arrows **G**–**H**)) and micro nuclei (stars **G**–**I**); Scale bar 10 µm. (**J**) percentage of embryos displaying phenotypes in the different matefin/SUN-1 strains: Large embryos (matefin/SUN-1 ^−/S43E^ *p* value = 1.5 × 10^−9^); mislocalized centrosomes (matefin/SUN-1 ^−/S43E^ *p* value = 8.6 × 10^−5^; matefin/SUN-1 ^+/S43E^ *p* value = 0.02); centrosome abnormalities (matefin/SUN-1 ^−/S43E^ *p* value = 0.01; matefin/SUN-1 ^+/S43E^ *p* value = 0.0001; matefin/SUN-1 ^−/S43A^ *p* value = 9.9 × 10^−5^; matefin/SUN-1 ^+/S43A^ *p* value = 0.0005) and micro nuclei (matefin/SUN-1 ^−/S43E^ *p* value = 0.04; matefin/SUN-1 ^+/S43E^ *p* value = 0.001; matefin/SUN-1 ^−/S43A^ *p* value = 0.001; matefin/SUN-1 ^+/S43A^ *p* value = 0.02); (**K**) matefin/SUN-1 S43E expression leads to disorganization of α-tubulin filaments in early embryos. Wild type matefin/SUN-1 embryos (**left**) and matefin/SUN-1 S43E embryos (**right**) stained with DAPI (**red**), anti SPD-5 (**blue**) and anti α-tubulin antibodies (**green**) during different stages of cell cycle. Scale bar 10 µm. Error bars represent SEM. * *p* < 0.05, ** *p* < 0.005, *** *p* < 0.0005 as compared to wild-type.

Previous studies showed that matefin/SUN-1 in combination with the KASH domain protein ZYG-12 mediates the proper localization of centrosomes to the nucleus [21,23,33]. Embryos expressing matefin/SUN-1 S43E in the null matefin/SUN-1 background had either reduced or no ZYG-12 staining (Figure 3). This result suggests that the dephosphorylated S43 matefin/SUN-1, but not the posphorylated S43 matefin/SUN-1, has a role in maintaining centrosome localization through its interaction with ZYG-12. Indeed, in 4.4% of S43E embryos in the presence of wild-type matefin/SUN-1 (*n* = 113) and in 7% in the absence of matefin/SUN-1 (*n* = 227) the centrosomes were detached from the nuclear envelope (arrowheads Figure 4C,D; quantified in Figure 4J). This phenotype was never observed in embryos expressing wild-type matefin/SUN-1::GFP (*n* = 87). In embryos expressing S43E matefin/SUN-1, the tight SPD-5 staining of the centrosome in wild-type embryos was not present (arrow in Figure 4A), and instead it was present in either a stretched or a diffused form (arrows in Figure 4E–I). This was evident both in the presence (34% of nuclei, *n* = 113) or absence (22% of nuclei, *n* = 227) of endogenous matefin/SUN-1 (Figure 4J) [22]. Staining of embryos expressing matefin/SUN-1 S43A with SPD-5 antibodies also showed abnormal centrosome structure including stretched centrosomes and disintegrated centrosomes. These phenotypes appeared in both the presence (29% of nuclei, *n* = 170) or absence (32% of nuclei, *n* = 147) of endogenous matefin/SUN-1 compared to only 11% (*n* = 87) in embryos expressing wild-type matefin::GFP (Figure 4J). However, the position of the centrosome next to the nuclear envelope was not significantly affected in these embryos, unlike in ZYG-12 mutations [23].

α-tubulin staining revealed that the abnormal centrosome position and organization in S43E embryos in matefin/SUN-1 null background were associated with abnormal microtubules organization [34] (Figure 4K).

The centrosome abnormalities can probably account for the micronuclei formation in S43 mutant embryos (stars in Figure 4G–I). Quantification analysis revealed the presence of micronuclei in 9% and 13% of S43A and 14% and 7% of S43E embryos in the presence or absence of wild type matefin/SUN-1, respectively. A phenotype that was specific to S43E in the absence of endogenous matefin/SUN-1 was the appearance of large size embryos (~2.3 times larger than wild type) in 15% of the 227 embryos analyzed (Figure 4B,J). These embryos also contained giant nuclei, which were probably formed by DNA endo-reduplication, nuclear fusion or both. Taken together, these results suggest that serine 43 in matefin/SUN-1 help regulate centrosome structure and localization to the nuclear envelope, as well as mitosis and embryonic size.

#### 3.1.4. Lamin Is Required for the Centrosome Localization of pS43.

**Figure 5:**
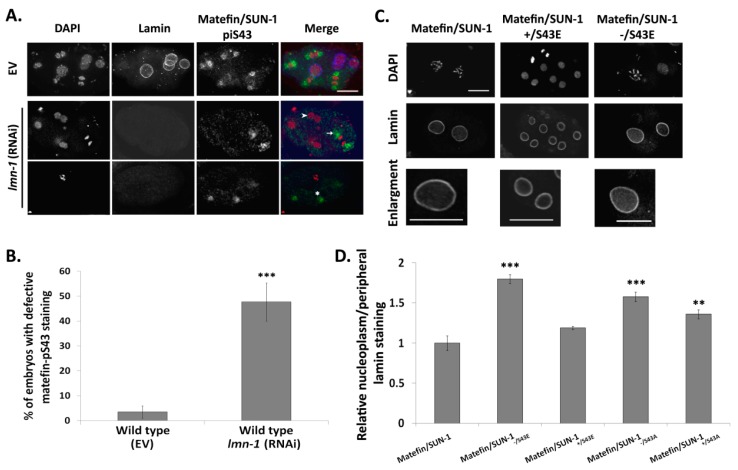
Matefin1/SUN-1 pS43 requires lamin for its proper localization. *lmn-1* (RNAi) disrupts matefin/SUN-1 pS43 localization in early *C. elegans* embryos. (**A**) wild type embryos stained with DAPI (red), anti ce-lamin (blue) and anti matefin/SUN-1 pS43 (green) antibodies. Top panel—embryo treated with EV; Bottom three panels—embryos treated with *lmn-1* (RNAi); (**B**) percentage of embryos displaying aberrant pS43 matefin/SUN-1 localization (*p* value = 1.07 × 10^−6^); (**C**) lamin localization was analyzed in embryos expressing matefin/SUN-1 S43 phosphorylation mutations. Embryos were stained with DAPI and anti ce-lamin antibodies; Scale bar 10 µm (**D**) quantification of the relative nucleoplasmic/peripheral ce-lamin staining in matefin/SUN-1 S43 mutant embryos. matefin/SUN-1 ^−/S43E^ *p* value = 6.6 × 10^−8^; matefin/SUN-1 ^+/S43E^ *p* value = 0.06; matefin/SUN-1 ^−/S43A^ *p* value = 1.3 × 10^−5^; matefin/SUN-1 ^+/S43A^ *p* value = 0.002. Error bars represent SEM. * *p* < 0.05, ** *p* < 0.005, *** *p* < 0.0005 as compared to wild-type.

Matefin/SUN-1 was previously shown to localize to interphase nuclei in a lamin independent manner [21]. In contrast, staining of *lmn-1* (RNAi) mitotic nuclei with pS43 antibodies revealed either lack of staining (arrowhead), aberrant localization (stars) or diffused staining (arrows) in ~50% of the nuclei (*n* = 44 Figure 5A,B).

We further analyzed lamin localization is embryos expressing S43 mutations, with and without endogenous matefin/SUN-1 by comparing the relative intensity of the nucleoplasmic lamin to the peripheral lamin. In S43A interphase embryos, nucleoplasmic lamin intensity was significantly increased both in the presence (36%, *n* = 19) or absence (58%, *n* = 22) of wild-type matefin/SUN-1 (Figure 5D). The most dramatic effect was observed in embryos expressing S43E in the absence of endogenous matefin/SUN-1, where there was an 80% (*n* = 39) increase in the relative intensity of the lamin staining in the nucleoplasm (Figure 5C,D). These results suggest a functional interaction between S43 in matefin/SUN-1 and lamin during mitosis.

**Figure 6:**
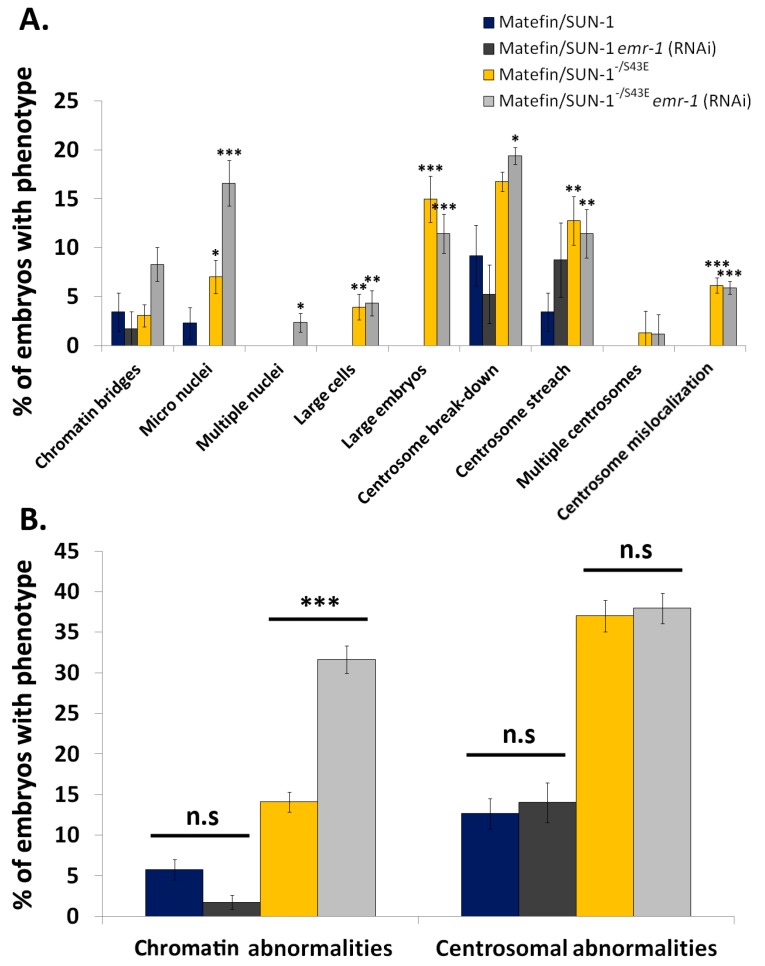
Emerin down regulation increases DNA abnormalities in a matefin/SUN-1 S43E embryos. DNA and centrosome abnormalities were analyzed in *emr-1* (RNAi) embryos expressing wild type or S43E matefin/SUN-1. (**A**) percentage of embryos displaying the phenotypes: chromatin bridges, micro-nuclei (S43E *p* value = 0.04; S43E *emr-1* (RNAi) *p* value = 8.3 × 10^−7^), multiple nuclei (S43E *emr-1* (RNAi) *p* value = 0.01), large cells (S43E *p* value = 0.002; S43E *emr-1* (RNAi) *p* value = 0.0008), large embryos (S43E *p* value = 1.5 × 10^−9^; S43E *emr-1* (RNAi) *p* value = 3.1 × 10^−8^), centrosome break-down (S43E *emr-1* (RNAi) *p* value = 0.01), centrosome stretch (S43E *p* value = 0.002; S43E *emr-1* (RNAi) *p* value = 0.005), multiple centrosomes and centrosome mislocalization (S43E *p* value = 0.0002; S43E *emr-1* (RNAi) *p* value = 8.8 × 10^−5^). *P* values as compared to wild type; (**B**) summary of results of chromatin and centrosome abnormalities from (**A**) the only significant result (*p* value = 2.9 × 10^−5^) was observed in S43E *emr-1* (RNAi). Error bars represent SEM. * *p* < 0.0 5, ** *p* < 0.005, *** *p* < 0.0005.

DAPI and SPD-5 staining of *emr-1* (RNAi) embryos expressing [10] wild-type matefin/SUN-1 revealed no affect of the treatment on chromatin or centrosome structure and localization ([10], and Figure 6). In contrast, *emr-1* (RNAi) embryos expressing S43E matefin/SUN-1 in a null matefin/SUN-1 background showed a significant increase in nuclei with abnormal chromatin (14% and 32%, *n* = 227 and 253, for control and S43, respectively). These phenotypes included chromatin bridges, micro nuclei, multiple nuclei and large cells, while centrosome phenotypes, which included centrosome break-down, centrosome stretch, multiple centrosomes and centrosome mislocalization, remained unaffected (37% and 38% for control and *emr-1* (RNAi), respectively) (Figure 6B; phenotypes depicted in Figure 4A–I). These results suggest that *emr-1* roles in mitosis are regulated through phosphorylation of serine 43 in matefin/SUN-1.

#### 3.1.5. Fertility Is Reduced in Animals Expressing S43E Matefin/SUN-1

**Figure 7:**
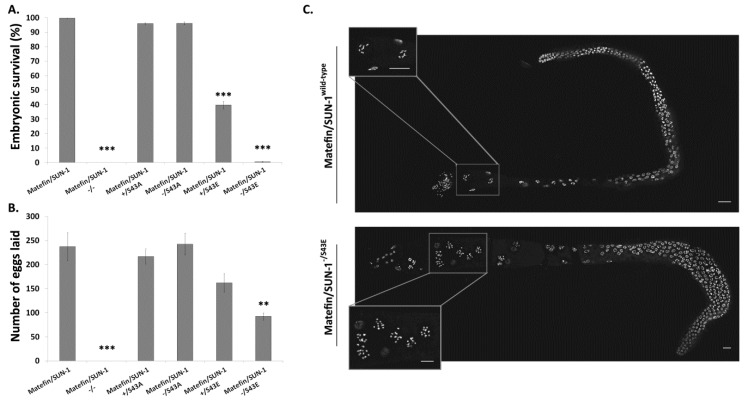
Constitutive expression of matefin/SUN-1 pS43 reduces animal fertility. Expression of S43E matefin/SUN-1 led to reduced: (**A**) embryonic viability. *p*-values: matefin/SUN-1 ^−/−^ = 1.74 × 10^−23^; matefin/SUN-1 ^+/S43E^ = 1.17 × 10^−8^; matefin/SUN-1 ^−/S43E^ = 1.18 × 10^−20^; (**B**) total number of egg laid. *p*-values: matefin/SUN-1 ^−/−^ = 2.7 × 10^−5^ and matefin/SUN-1 ^−/S43E^ = 0.003. Error bars represent SEM. * *p* < 0.05, ** *p* < 0.005, *** *p* < 0.0005 as compared to wild-type; (**C**) DAPI staining of gonads taken from animals expressing wild type matefin/SUN-1:GFP (**top panel**) and matefin/SUN-1 ^−/S43E^ (**bottom panel**). Matefin/SUN-1 pS43 expression prevents the formation of bivalents during diakinesis. Enlarged boxes show diakinesis nuclei. Scale bar 10 µm.

Loss of matefin/SUN-1 during larval and adult stages causes animal sterility [21,22] (Figure 7A,B). Animals expressing the S43A mutation either in the presence or absence of endogenous matefin/SUN-1 had a similar percentage of viable embryos (96% for both) and number of laid eggs (241 and 243, respectively) to that of wild-type animals (98%, 238) (Figure 7A,B). In contrast, animals expressing the S43E mutation showed reduced offspring viability (39.8%) in the presence of endogenous matefin/SUN-1 and almost complete absence of viable offspring in its absence (0.6%) (Figure 7A). The number of laid eggs of animals expressing the S43E mutation was reduced by 39% in the absence of endogenous matefin/SUN-1 (Figure 7B). The later results suggested that expression of S43E prevents the normal progression of meiosis. Indeed while DAPI analysis of gonads of wild-type and S43A animals showed six bivalent chromosomes in diakinesis gonads (Figure 7C top panel *n* = 5). In animals expressing S43E in a matefin/SUN-1 null background in diakinesis, nuclei had 11–12 univalent chromosomes (Figure 7C bottom panel). The inability of S43E animals to form bivalents can explain the lack of viable embryos.

Together, these results suggest that correct S43 phosphorylation of matefin/SUN-1 is required for both the normal progression of meiosis, as well as mitosis during early embryogenesis.

### 3.2. Discussion

Matefin/SUN-1 is a *C. elegans* inner nuclear membrane protein that is present only in germ cells and early embryos. Previous studies demonstrated a role for matefin/SUN-1 in germline development, attaching centrosomes to the nucleus and homologous chromosome pairing during meiosis. In this study, we show that the phosphorylated form of the serine 43 in the N-terminal domain of matefin/SUN-1 (S43) is predominantly localized to centrosomes (Figure 1B) where it is phosphorylated in a CDK-1 dependent manner (Figure 2). These results are in agreement with a recent publication showing that three mitotic phosphorylation sites exist in the human SUN1 N-terminal nucleoplasmic domain (S48, S138 and S333), two of which are also phosphorylated by CDK-1 [35].

It was previously shown that matefin/SUN-1 and the KASH domain protein ZYG-12 are required for centrosome attachment to the nucleus in early embryos [23,33]. Mimicking constitutive phosphorylation of serine 43 in matefin/SUN-1 in embryos, led to either loss or reduced amounts of ZYG-12 at the nuclear periphery, as well as to dissociation of a fraction of centrosomes from the nuclear envelope (Figure 3 and Figure 4C,D,J). These data suggest that both ZYG-12 and centrosome localization are mediated through the dephosphorylated form of S43 in matefin/SUN-1. Centrosomes are essential during metaphase for proper mitotic spindle formation and spindle positioning but dispensable during anaphase [36]. Here, we show that, in addition to centrosome tethering to the nucleus, aberrant matefin/SUN-1 S43 phosphorylation perturbs centrosome structure and α-tubulin organization in a dominant manner (Figure 4E–K). These results can have implication to Emery–Dreifuss muscular dystrophy (EDMD) due to the fact that a recent report shows that disease associated mutations in SUN-1 impair microtubule organization and centrosome attachment to the nuclear envelope in EDMD patient myotubes [37].

Although the interphase localization of matefin/SUN1 does not require lamin, and interphase lamin localization does not require matefin/SUN-1, the two proteins directly interact with each other *in vitro* [21] and are required for each other’s localization during mitosis (Figure 5); in early *C. elegans* embryos, constitutive phosphorylation of S43 in matefin/SUN-1 resulted in 80% increase in relative lamin intensity in the nucleoplasm and pS43 localization to centrosomes during cell cycle required lamin. This functional interaction between lamin and pS43 could be conserved in evolution since the nucleoplasmic tail of mammalian SUN1 binds lamins A/C [38], it loses this interaction during mitosis, and SUN1 is specifically phosphorylated by CDK-1 at position S48 [35].

In myoblasts of EDMD patients carrying SUN-1 mutations, emerin transcripts are reduced and emerin interaction with SUN-1 is impaired [37]. Our data show that regulation of *emr-1* in S43E matefin/SUN-1 expressing animals led to a significant increase in chromatin phenotypes, while the position and structure of centrosomes were not further altered (Figure 6). In *C. elegans*, *emr-1* by itself is not an essential gene and only its loss in combination with another LEM domain gene, *lem-2*, leads to an increase in the occurrence of anaphase bridges and condensed chromatin [10]. Our results suggest that matefin/SUN-1 regulates emerin’s mitotic roles either directly through its S43 phosphorylation, or indirectly through its effect on lamin localization.

In human laminopathies, SUN-1 and SUN-2 act as disease modifiers, aggravating disease phenotypes in EDMD patients. In addition, reduction in SUN-1 levels in HGPS patient fibroblasts improved nuclear phenotypes and reduced the cellular senescence of these cells [39]. Understanding the different roles of SUN-domain proteins will enable better understanding of the disease mechanisms of both EDMD and HGPS.

## Supplementary Materials

The following are available online at: http://www.mdpi.com/2073-4409/5/1/8/s1, Figure S1: Matefin/SUN-1 piS8 and pS24 localization during cell cycle, Figure S2: RNAi screen of potential kinases involved in matefin/SUN-1 S43 phosphorylation.
