# Supplementary material for: Matefin/SUN-1 Phosphorylation on Serine 43 Is Mediated by CDK-1 and Required for Its Localization to Centrosomes and Normal Mitosis in C. elegans Embryos
Source: Cells. 2016 Feb 24;5(1):8. doi: 10.3390/cells5010008 (PMC4810093; doi:10.3390/cells5010008)
Supplement: Supplementary file 1 [file cells-05-00008-s001.pdf]

Matefin/SUN-1 Phosphorylation on Serine 43 is Mediated by CDK-1 and Required for Its Localization to Centrosomes and Normal Mitosis in *C. elegans* Embryos

Noam Zuela and Yosef Gruenbaum

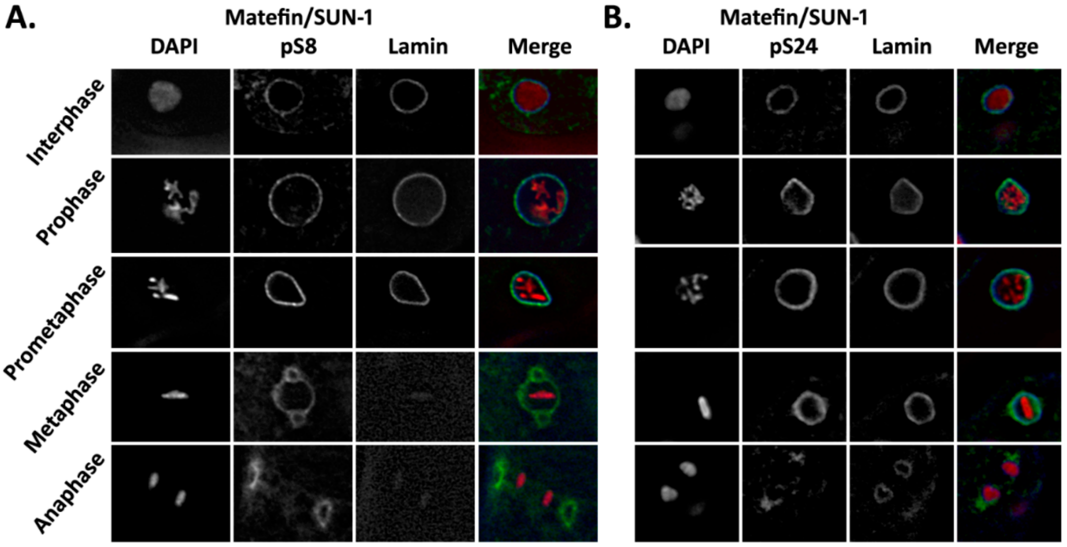

Figure S1. Matefin/SUN-1 piS8 and pS24 localization during cell cycle. (**A**) Matefin/SUN-1 pS8 and (**B**) Matefin/SUN-1 pS24 localization during different stages of cell cycle. Embryos stained with DAPI (red), anti ce-lamin (blue) and anti matefin/SUN-1pS8/24 (green) antibodies. Scale bar 10 µm.

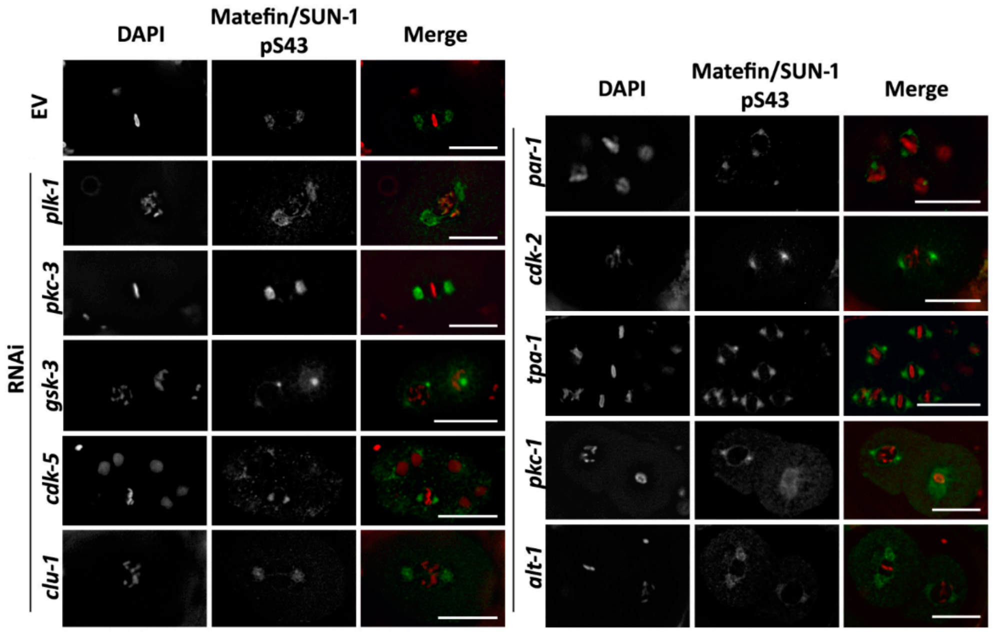

Figure S2. RNAi screen of potential kinases involved in matefin/SUN-1 S43 phosphorylation. *C. elegans* early embryos were subjected to RNAi treatment against different kinases and stained with DAPI (red) and anti matefin/SUN-1pS43 antibodies (green). Top left panel: Embryos treated with EV; Bottom left panels and right panels: Embryos treated with RNAi of different kinase genes (*plk-1*, *pkc-3*, *gsk-3*, *cdk-5*, *clu-1*, *par-1*, *cdk-2*, *tpa-1*, *pkc-1* and *alt-1*). No effect on pS43 staining was detected. Scale bar 10 µm.
